# Key Physical Factors in the Serve Velocity of Male Professional Wheelchair Tennis Players

**DOI:** 10.3390/ijerph18041944

**Published:** 2021-02-17

**Authors:** Alejandro Sánchez-Pay, Rafael Martínez-Gallego, Miguel Crespo, David Sanz-Rivas

**Affiliations:** 1 Department of Physical Activity and Sport, Faculty of Sport Sciences, University of Murcia, C/Argentina, s/n, 30720 San Javier, Spain; 2 Department of Physical Education and Sport, Faculty of Sport Sciences, University of Valencia, Av. Blasco Ibáñez, 13, 46010 Valencia, Spain; rafael.martinez-gallego@uv.es; 3 International Tennis Federation, London SW15 5XZ, UK; Miguel.crespo@itftennis.com; 4 Tennis Research Group, 28080 Madrid, Spain; dsanzrivas@gmail.com

**Keywords:** tennis, movement, biomechanics, physical tests

## Abstract

The aim of this study was to identify the physical factors related to serve speed in male professional wheelchair tennis players (WT). Nine best nationally-ranked Spanish male wheelchair tennis players (38.35 ± 11.28 years, 63.77 ± 7.01 kg) completed a neuromuscular test battery consisting of: isometric handgrip strength; serve velocity; 5, 10 and 20 m sprint (with and without racket); agility (with and without racket); medicine ball throw (serve, forehand and backhand movements); and an incremental endurance test specific to WT. Significantly higher correlations were observed in serve (r = 0.921), forehand (r = 0.810) and backhand (r = 0.791) medicine ball throws showing a positive correlation with serve velocity. A regression analysis identified a single model with the medicine ball throw serve as the main predictor of serve velocity (r^2^ = 0.847, *p* < 0.001). In conclusion, it is recommended that coaches and physical trainers include medicine ball throw workouts in the training programs of WT tennis players due to the transfer benefits to the serve speed.

## 1. Introduction

Wheelchair tennis (WT) is the adapted modality of conventional tennis (CT) [1]. WT is one of the most popular Paralympic sports [2]. This progress has increased the competitive level of the players and has driven the professionalization of the best-ranked ones [3]. In order to assist WT players in their quest for performance improvement and professionalization, creating specific training situations that simulate the reality of the competition has been indicated as a key factor in the design of the sessions [4]. For this, it is vitally important for coaches and physical trainers to better understand the factors that specifically affect WT performance in order to achieve the best results.

WT competition is divided into two categories: Open and Quad. In the Open category, there are two draws: women and men [1]. In this category players have a wide range of disabilities, including spinal cord injury, single amputees, double amputees or spina bifida. In the Quad category, men and women play together and they also have a disability in their upper limbs as well [5].

**Figure 1:**
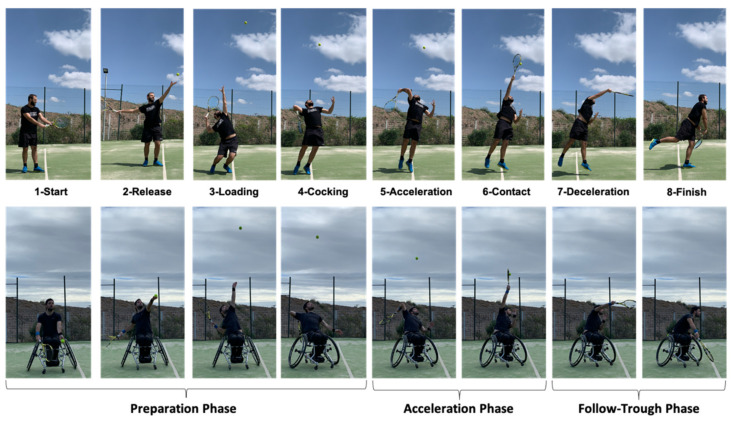
Service sequence in conventional and wheelchair tennis.

Most of the studies conducted on WT match analysis have focused on the Open category. These research have concluded that WT rally length has been shown to last between six to ten seconds, with three to four shots per rally [6,7]. Serve and return of serve seem to be the most important strokes in a WT match. In conventional tennis (CT) the serve has been described as the most potentially dominant stroke in the modern game [8,9], although in WT it does not seem to have the same positive influence than in TC [10,11]. Serve velocity (SV) is undoubtedly one of the determining factors for standing players [12] and its relationships with other factors related to the physical condition of the players has been widely studied [13]. Some research has used different isometric tests, such as wrist, elbow or shoulder flexion-extension [14], whereas other studies have used dynamic strength tests such as the isokinetic shoulder test [15] to find the relationships between physical condition and service speed. On the other hand, studies have also used functional field tests related to medicine ball throwing as possible predictors of service speed [13,16,17]. In general, it seems that knee flexion before extension is a prerequisite for an efficient execution of the serve as well as to achieve a higher jump [18,19] aspects that cannot be considered in adapted tennis because it is played sitting on the wheelchair (Figure 1).

Some studies conducted by national tennis associations have used a battery of physical tests to know the evolution of their athletes [20,21], as well as to establish relationships between the measurements [13,22,23]. In general, anthropometric measurements, strength, speed, agility, endurance and flexibility are usually included in these tests.

From a biomechanics perspective, the serve movement is commonly divided into three phases (preparation, acceleration and follow-through) including eight stages (start, release, loading, cocking, acceleration, contact, deceleration and finish) [24]. The loading stage of the lower body has been described as the ‘loaded position’ the dominant elbow adopts at its lowest vertical position, it coincides with the maximal knee flexion [24] and it occurs at the end of the eccentric phase of the movement. In the case of WT, the players have a lower hitting plane as compared to the standing players, as well as a lower force generation due to the deficit in the production of force of the lower body [25]. In addition, the functional limitation of the WT players implies that the Quad category players, who have a high functional limitation, impact the ball closer to the body and generate a lower hitting power than those of the Open category [26]. An increasing serve speed reduces the time for the opponent to successfully return the ball and increases the probability of the server’s dominance in the rally or of winning a direct point [27]. Due to the fact that, in CT, there are studies that show a relationship between physical parameters and service speed [13,14,15,16], and that the service technique is similar between both disciplines, our hypothesis is that there will be a relationship between some physical parameters and the service speed in WT players. Despite this, to the authors’ knowledge there is no research on how the serve speed is related to the WT athlete’s physical parameters, where field tests have become a reliable option to establish the performance level of players [28]. Therefore, the objective of this research was to identify the physical factors related to the velocity of the serve in WT players using different reliable and valid field tests previously utilized in research.

## 2. Materials and Methods

### 2.1. Participats and Procedures

Nine of the top ten male wheelchair tennis players in the Spanish national ranking participated in this research (mean ± SD age: 38.35 ± 11.28 years, weight: 63.77 ± 7.01 kg). All of them played national and international competitions and were among the top 150 international WT ranking ITF (Open category). Eight of the nine players were right-handed, and one was left-handed. Players had 10.2 ± 6.2 years of playing experience and practised an average of 9.3 ± 4.8 hours of tennis per week. The characteristics of the participants are shown in Table 1. The players were informed of the characteristics of the study and signed an informed consent to participate in it. All the procedures followed in this study were in accordance with the ethical standards of the Declaration of Helsinki of 1975, revised in 2008, and were approved by the ethics committee of the Royal Spanish Tennis Federation (RFET_CE17.3).

The players were summoned at the same time of day to perform the tests [29]. First, a standardized 10-min directed warm-up was performed consisting of joint mobility, linear movements with the chair, circular movements and turns simulating hitting, and low-intensity accelerations and decelerations [30]. The tests were carried out on two consecutive days in the following order: Day 1: Sprint test (5, 10 and 20 m), agility test (T-test), service speed test, and medicine ball throw test (forehand, backhand and serve); Day 2: Incremental resistance test (Hit and Turn Tennis Test) and manual dynamometry test. The scores of the different tests were collected during their development by the researchers themselves. All tests were conducted on an indoor hard tennis court.

### 2.2. Measurements Collected

**Figure 2:**
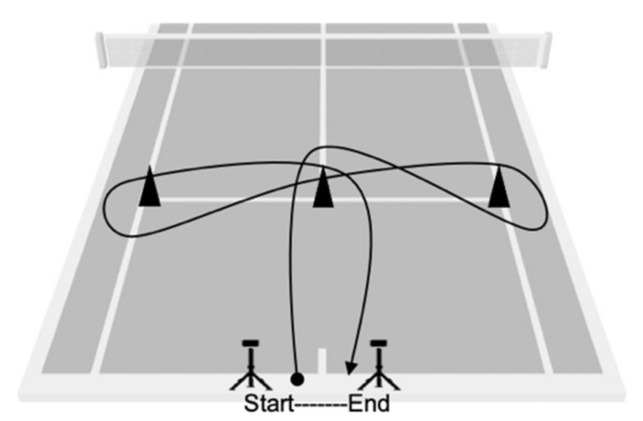
Agility test (T-Test).

**Figure 3:**
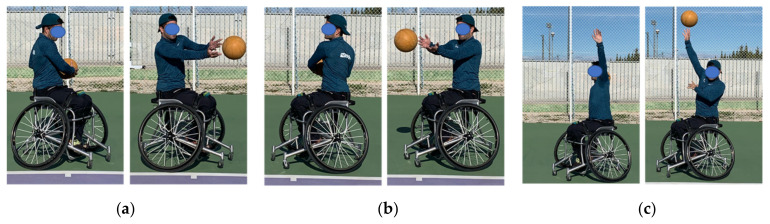
Medicine ball through backhand (**a**), forehand (**b**), and serve (**c**).

Different reliable and valid field tests used previously in research were selected. The characteristics of each of the tests were the following [28,30,31,32]:

- Sprint test: Four gates at 0, 5, 10 and 20 m were used to measure the speed of the WT players. Subjects started from a line 0.5 m behind the first gate. Each participant performed the test three times without a racket, and three times with a racket, with a 2 min rest time between each repetition. The best value of the three attempts was recorded. The time was counted in seconds (s) and thousandths of a second (ms) with an error of ± 0.001 s through Chronojump photocell^®^ (Chronojump, Barcelona, Spain) and Chronojump software version 1.7.1.8 (Chronojump, Barcelona, Spain) for MAC.
- Agility test (T-Test): This agility test is adapted for wheelchair sports [31] and has previously been used in WT players [33]. The test includes accelerations and decelerations, as well as turns for both sides. The participant started in the centre of the court behind the baseline, they had to move to the intersections of the singles line with the service line, always passing through the central area of the court (T) until returning to the starting area (Figure 2). Each participant performed the test three times without a racket, and three times with a racket, with a 2 min rest time between each repetition. The best value of the three attempts was recorded. Time was measured using the Chronojump Photocell^®^ (Chronojump, Barcelona, Spain) and the Chronojump software version 1.7.1.8 for MAC with a gate located on the baseline to record the start and the end of the test.
- Serve velocity test: A radar gun (Stalker Pro Inc., Plano, Texas, USA) was used to measure serve velocity. The player performed 10 services at maximum velocity directed to the wide area of the service box from the advantage side for the right-handed players, and from the deuce side for the left-handed player [34]. The radar was positioned behind the player at the same hitting height and oriented in the same direction as the ball. The average value of 10 serves in km·h^−1^ was recorded.
- Upper body strength: Explosive strength was evaluated through three medicine ball through tests, simulating the forehand, the backhand and the serve shots [35,36]. The participants stood behind the throwing line in a 45° position. A 15 m long measuring tape was placed on the court perpendicular to the throwing line and two evaluators marked the bounce zone of the recorded ball in 0.10 m sections. A 2-kilogram medicine ball was used for the test. Participants performed each type of throw three times, with a 2 min rest time between each repetition. The players had to throw the ball simulating the technical gesture of the backhand (Figure 3a), the forehand (Figure 3b) and the serve (Figure 3c).
- Isometric handgrip strength: The hand dynamometry test was carried out to assess the maximum isometric force in the flexors of the fingers with a Smedley III T-18A dynamometer (Takei, Tokyo, Japan) and a range between 0 and 100 kg in 0.5 kg increments and an accuracy of ±2 kg. The test was carried out in the wheelchair sitting position with the arm extended and glued to the wheel without actually contacting it [30]. Each subject made three maximum attempts with each hand after a familiarization phase with the instrument with sub-maximum repetitions. The rest time between each attempt was 2 min. The best value of three attempts was recorded in N·kg^−1^.
- Anaerobic endurance test (Hit and Turn Tennis Test): This test is an adaptation of the one developed for conventional tennis to evaluate the specific anaerobic endurance of the player through the level reached [32]. The test consists of simulating a hit on top of a cone located at the intersection of the doubles line with the baseline line, coinciding with the sound signals emitted by the sound of the test. After that hit, the player must simulate another hit on the opposite side and so on until the end of the series. In this adaptation, the hitting had to be made close to a cone located between the intersection of the singles line with the doubles line, thus reducing the distance of displacement for the WT players. As an incremental test, it ended when the player was unable to reach the cone at the rate set by the sound signals. The period reached by each player was recorded when he was no longer able to simulate hitting in the designated area at the same time as the acoustic signal sounded.

Table 2 shows the physical variables measured as well as the different tests used.

### 2.3. Data Analysis

Due to the small size of the sample, the Shapiro–Wilk and Levene tests were used to contrast the normality and homogeneity of variances for each variable (sprint, agility, strength and anaerobic endurance). All the variables obtained *p*-values > 0.05 except in the dynamometry with the dominant arm. A Pearson correlation analysis (Kendall’s Tau-b for dominant dynamometry) was performed to identify those variables related to serve speed. Values were classified as trivial (0–0.1), small (0.1–0.3), moderate (0.3–0.5), large (0.5–0.7), very large (0.7–0.9), almost perfect (0.9) and perfect (1.0) [37]. Subsequently, a multiple linear regression analysis (stepwise) was performed to identify the parameters with the greatest influence on SV. The SV was used as a dependent variable, while the rest of the variables that had previously shown significance operated as independent. Significance was established at *p* < 0.05. All data were analyzed with the IBM SPSS 25.0 statistical package for Macintosh (IBM Corp, Armonk, NY, USA).

## 3. Results

Table 3 shows the descriptive analysis of test measurement in wheelchair tennis players.

**Figure 4:**
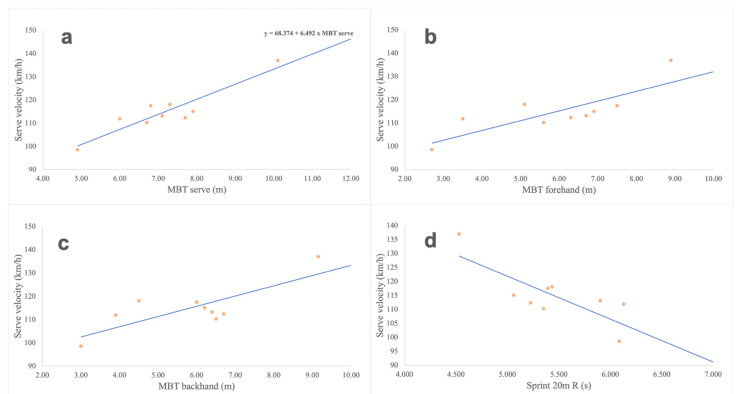
Relationship between the medicine ball throw test simulating a serve (**a**), forehand (**b**), backhand (**c**) and sprint of 20 m with racket (**d**) with the serve velocity (km/h).

Table 4 shows the correlation coefficients of the different physical tests performed with the serve speed. Figure 4 shows the relationship between statistically significant variables and serve velocity. Significantly higher correlations were observed in medicine ball throws for service (r = 0.921), forehand (r = 0.810) and backhand (r = 0.791) showing a positive correlation. The 20-m racket test showed significance (*p* = 0.012) and negatively correlated with serve velocity (r = −0.788).

Table 5 shows the results of the multiple regression analysis. The medicine ball throw simulating a serve was shown as the main and only predictor measure of the speed of the serve (r^2^ = 0.847, *p* < 0.001) with a positive relationship.

## 4. Discussion

Knowing how the physical demands of WT are related, as well as identifying which are the variables that determine any of them, can help coaches and physical trainers in the design of exercises adapted to the specific needs of the game. The aim of this research was to determine the relationship of different physical demands evaluated through a field test battery with the serve velocity in professional WT players. In general, it was observed that medicine ball throws simulating the forehand, the backhand and the serve strokes showed the highest correlation with SV, while the serve medicine ball throw was the test that best predicted the model.

Coaches and physical trainers often use batteries of tests (related to speed, agility, maximum strength or functional movements) to understand the evolution of their athletes and to prescribe training, among other goals [38]. Medicine ball throws both in the forehand and the backhand showed a positive and statistically significant correlation with service speed (Table 4). These throws have been useful to examine the rotational power of the trunk of athletes in general [39] and in tennis players in particular [38].

The tennis serve includes the activation of the abdominal muscles (rectum and obliques) to perform a trunk flexion with a rotation [25]. Furthermore, the forehand MBT had a higher correlation with serve velocity than the backhand MBT (r = 0.810 vs. r = 0.791). It is worth noting that the forehand MBT includes a rotation of the trunk towards the player’s dominant side, as does the serve, which could explain the greater correlation between both. In addition, the 20-m sprint showed a negative correlation with service speed (r = −0.788). In this sense, a greater throw distance is related to a higher displacement speed (less time) over long distances (20 m) (Figure 4d). The abdominal muscles have a great implication in the generation of force in the service [25], and also in the stabilization of the trunk for the propulsion tasks [40], which could explain the observed correlation.

The biomechanical requirements of the serve have been specifically analysed using an 8-stage model (star, release, loading, cocking, acceleration, contact, deceleration and finish) [24]. Due to the considerably low contribution of the lower body to generate force in the kinetic chain of the service movement in WT players, it could be indicated that from the loading phase (semi-side position, elbow of the racket arm at its lowest position, free arm stretched up, etc.) the movement of the upper body in the serve is similar to the service MBT in both standing and chair players. This could explain that the medicine ball throw simulating a serve is the main variable predicting the speed of serve (Table 5) showing a high correlation (Figure 4a). In addition, this loading position in both shots has specific biomechanical implications that do not occur in the forehand and the backhand MBT (shoulder over shoulder work, action-reaction of free arm, line of force in the direction of throw/impact, asynchronous movements between both arms, etc.) [41,42].

In fact, it is known that one of the movements that generates greater power in the hitting action is the torque or rotation component of the trunk to increase the acceleration distance with respect to the point of impact, as well as to add a greater number of elements of the kinetic chain in the corresponding stroke action [43]. Therefore, this action is very clear in the forehand and backhand strokes, but it also has a very relevant role in the serve movement. In this study, it has been observed that from a kinematic point of view, actions that resemble the forehand, the backhand and the serve strokes have a high correlation with serve speed (Figure 4a–c). Specifically, and from a kinematic point of view, the action of MBT serve reproduces the serve movement in its setup and advance-impact phases (Figure 1). Even wheelchair tennis players who have a spinal cord injury and functional deficit in the trunk musculature, can use their non-dominant hand as a support in the hitting action, in a similar way to that which occurs in the serve movement of standing players. In fact, the serve requires multiarticular recruitment and a high rotation speed during the shot [41,42].

Due to the fact that the serve and, specifically, the SV have been reported as the most powerful and dominant stroke feature in tennis, involving various factors such as the strength and power of the upper body and the range of motion of the shoulder [24]; and without considering other elements of the kinetic chain such as the lower body (which also has its influence), it can be considered that this stroke is also decisive in WT. Therefore, and as per the results obtained, it seems reasonable to think that specific strength training prescription, simulating the kinematics of the serve gesture with medicine balls, can help to achieve speed increases especially in the serve, and also in the forehand and backhand strokes. Therefore, it is recommended that coaches and physical trainers incorporate this type of specific work in their training plans to assist players in improving these strokes. These workouts should be done in neuromuscular working conditions, in the absence of fatigue and at the beginning of the training sessions, to avoid the practice of serve stroke workouts in the final parts of the training sessions as usual.

The results obtained in this study present a series of limitations that should be considered. On the one hand, although the sample includes the top nine national players in the Open category, the small size of the sample does not allow for divisions according to their functional limitation, since the strength of the relationship between the variables would have been very light. Therefore, future research should include a larger sample of WT players, players from different WT categories (Quad and Open), as well as female and male players to compare this relationship (SV with physical tests), both due to functional limitation and between genders. In addition, other anthropometric variables, such as height or body segments, were not measured, which have shown a correlation with service speed in CT players. On the other hand, the SV test was carried out in a training environment and taking measurements in a competition setting using a radar gun could provide different data.

## 5. Conclusions

Medicine ball throws simulating the forehand, the backhand and the serve strokes showed a high correlation with SV in WT. The MBT serve is the test that best predicts SV in WT. Therefore, and given the similarity of the movements between the two gestures, coaches and physical trainers are encouraged to include medicine ball throws workouts as a service transfer exercises within the training programs of WT players. Likewise, it is advisable to work with medicine ball throws for the forehand and backhand, given the importance of trunk rotation for the serve.

## Figures and Tables

**Table 1:** Characteristics of the sample of wheelchair tennis players participating in the study.

*n*	National Ranking	International WT Ranking (ITF)	Age	Weight (kg)	Playing Training hours per week	WT Playing Experience (years)
1	1	Top 15	24	61	20	12
2	2	Top 15	18	65	15	8
3	3	Top 40	34	67	8	5
4	4	Top 50	45	57	3	24
5	5	Top 60	39	52	6	9
6	6	Top 100	35	72	10	2
7	8	Top 100	50	73	8	17
8	9	Top 150	50	70	6	8
9	10	Top 150	41	57	8	7

**Table 2:** Physical variable and test event.

Physical Variable	Test	Characteristic
Strength	Grip strength (2)	1-Dominant 2-Non-Dominant
	Medicine ball through (3)	1-Forehand 2-Backhand 3-Serve
	Serve velocity	Average value of 10 serves
Sprint	5 m	With and without racket
	10 m	
	20 m	
Agility	T-Test	With and without racket
Endurance	Hit and Turn Tennis Test	With racket

**Table 3:** Mean (M), standard deviation (SD) and confidence interval (CI) of physical test measurements.

Test	M	SD	CI 95%
Grip strength. Dom. (N·kg^−1^)	46.33	4.13	43.11;49.54
Grip strength. No Dom. (N·kg^−1^)	38.61	6.26	33.79;43.43
Service velocity (km·h^−1^)	114.90	10.06	107.16;122.63
Sprint 5m NR (s)	1.555	0.16	1.42;1.68
Sprint 10m NR (s)	2.977	0.27	2.76;3.18
Sprint 20m NR (s)	5.403	0.50	5.01;5.78
Sprint 5m R (s)	1.657	0.21	1.49;1.82
Sprint 10m R (s)	3.021	0.37	2.73;3.31
Sprint 20m R (s)	5.456	0.51	5.05;5.85
T-Test NR (s)	12.426	0.99	11.66;13.19
T-Test R (s)	12.681	1.20	11.75;13.6
MBT F (m)	5.91	1.93	4.42;7.4
MBT B (m)	5.81	1.81	4.42;7.21
MBT S (m)	7.16	1.42	6.06;8.26
Hit and Turn (n)	15.22	2.99	12.92;17.52

Dom: Dominant. No Dom.: Non-dominant. NR: no racquet. R: racquet. MBT: medicine ball throw. F: Forehand. B: Backhand.

**Table 4:** Correlation coefficient of physical tests with serve velocity.

Test	r	*p*
Grip strength. Dom. (N·kg^−1^)	0.582	0.100
Grip strength. No Dom. (N·kg^−1^)	0.297	0.438
Sprint 5m NR (s)	−0.613	0.079
Sprint 10m NR (s)	−0.518	0.154
Sprint 20m NR (s)	−0.523	0.148
Sprint 5m R (s)	−0.502	0.168
Sprint 10m R (s)	−0.599	0.089
Sprint 20m R (s)	−0.788	0.012
T-Test NR (s)	−0.623	0.073
T-Test R (s)	−0.585	0.098
MBT F (m)	0.810	0.008
MBT B (m)	0.791	0.011
MBT S (m)	0.921	< 0.001
Hit and Turn (n)	0.608	0.082

Dom: Dominant. No Dom.: Non-dominant. NR: no racquet. R: racquet. MBT: medicine ball throw. F: Forehand. B: Backhand. S: Serve.

**Table 5:** Statistics of multiple regression analysis.

	R	R^2^	R*^2^* Adjust	F	Sig F.	Regression Equation
Model	0.921	0.847	0.826	38.953	<0.001	
			Beta	T	Sig T.	y = 68.374 + 6.492 × MBT S
MBT S			0.921	6.241	<0.001	

MBT: medicine ball throw. S: Serve.

## Data Availability

The data presented in this study are available on request from the corresponding author. The data are not publicly available due to privacy.
